# Contributions of Individual, Family, and School Characteristics to Chilean Students’ Social Well-Being at School

**DOI:** 10.3389/fpsyg.2021.620895

**Published:** 2021-02-26

**Authors:** Verónica López, Javier Torres-Vallejos, Paula Ascorra, Luis González, Sebastián Ortiz, Marian Bilbao

**Affiliations:** ^1^School of Psychology, Pontificia Universidad Católica de Valparaíso, Valparaíso, Chile; ^2^Center for Research in Inclusive Education, Valparaíso, Chile; ^3^Universidad Andres Bello, Santiago, Chile; ^4^School of Psychology, Universidad Alberto Hurtado, Santiago, Chile

**Keywords:** social well-being, school, school climate, family, multi-level

## Abstract

Schools are an essential part of students’ lives and can promote and facilitate their well-being. Although research on well-being among school-aged children and adolescents has distinguished subjective well-being from social well-being, very few studies examined student’s social well-being at school (SWS). SWS is understood as students’ valuation of the circumstances and functioning of their school. This framework posits that the context of the schools can shape students’ perception of feeling integrated and making significant contributions to their schools. However, not much is known regarding the joint contribution of individual, family, and school characteristics to students’ SWS. This study analyzed these joint contributions in a nationally representative sample of 6,389 children and adolescents enrolled in 5th–11th grades. Findings show that being female and younger were individual predictors of SWS. Students’ satisfaction with their family and fewer changes of schools were also significant contributors. When students’ perceptions of their schools were incorporated, the individual and family characteristics decreased or lost significance. In the full model, the highest contribution to SWS was explained by the school-level aggregated perception of school climate. These findings call for integrated policies and practices to foster students’ sense of belonging, feeling integrated, and contribution to their schools, with a focus on school-level interventions to improve SWS through positive and engaging school climates that foster students’ sense of agency.

## Introduction

Schools are an essential part of students’ lives and can promote and facilitate their well-being. Research on well-being among school-aged children and adolescents has distinguished subjective well-being from social well-being. Whereas subjective well-being refers to how people experience and evaluate their lives in general and in specific domains (Krueger, 2009), social well-being refers to how they evaluate the quality of their relationships with other people in general and in specific contexts (Keyes, 2006, p. 5). In this study, we posited that social well-being at school (SWS) can be understood as students’ valuation of their school’s circumstances and functioning, acting as a context-based evaluation of their quality of life in society (Keyes, 2009). School is an important domain of society for children and adolescents due to the amount of time spent in schools and the multiple opportunities for engaging in significant social interactions with peers and adults that attending school implies. Therefore, a sense of belonging and contributing to their schools is an important aspect of children and adolescents’ quality of life. Based on the social well-being framework (Keyes, 2009), we posited that the contexts of schools can shape students’ perception of feeling integrated and making significant contributions to their schools. In this sense, schools can make a difference in shaping students’ SWS. As an essential part of students’ lives, schools can promote and facilitate their well-being, but they can also hinder it depending on the opportunities for enriched social and pedagogical interactions they offer to all students. However, it is also possible that factors outside the school context, such as the social and economic circumstances that shape students’ individual experiences and their family experiences, may also contribute to their SWS. However, there is scarce research regarding the joint contribution of individual, family, and school characteristics to students’ SWS (Ahmadi and Ahmadi, 2019). Hence, this study analyzed these joint contributions in Chilean students.

In recent years, the study of well-being has become a challenge for school systems, because of not only its impact on the mental health of students (Berger et al., 2009; Cobo et al., 2020) but also its contribution to improving academic results (López et al., 2017; Govorova et al., 2020). Students who perceive themselves as integrated, accepted, and contributing to their context and that their context is favorable and consistent with their own needs (Keyes, 1998; Keyes and Shapiro, 2004) present not only better adjustment in individual psychological variables and mental health (Keyes, 2013; Venning et al., 2013) but also better results on performance measurement tests. Research has also shown that higher levels of well-being improve students’ average school attendance and with it, indicators of motivation and commitment to learning (Oyarzún, 2018), preventing dropout and dropout-related phenomena (Torres-Vallejos, 2020). Likewise, its consideration has been brought to the fore in the reduction of violent behaviors among peers (Berger et al., 2009; Benbenishty and Astor, 2018), with fewer punitive practices associated with school suspensions and expulsion (Norris, 2018). Therefore, schools are an essential promoter of individual and social well-being for students.

In this study, we argue for the need to approach the study of students’ well-being from a more complex and socioecological approach (Astor and Benbenishty, 2018), which allows integrating the individual–environment dialectic considering not only the individual voices of participants (Oyarzún et al., 2017) but also the joint contribution of individual and contextual factors (Fernández et al., 2020).

Social well-being is defined as “individuals’ perceptions of the quality of their relationships with other people, their neighborhoods, and their communities” (Keyes, 2006, p. 5). This construct is made up of five subdimensions: (a) social integration: an individual appreciation of the quality of our relationships with society and the community; (b) social acceptance: trust in others and acceptance of the positive and negative aspects of our life; (c) social contribution: a feeling of usefulness and being able to contribute something to the society in which we live; (d) social actualization: confidence in the future of society and its ability to produce conditions that favor well-being; and (e) social coherence: confidence in the ability to understand the dynamics and functioning of the world in which we live. The literature on social well-being emphasizes that it contributes to the construction of a life with meaning and purpose by having meaningful relationships with others and feeling that we belong in a relevant way to the social and surrounding environment, allowing us to develop and contribute to an imagined future (Blanco and Díaz, 2005; Keyes, 2009; Bilbao et al., 2014). According to Cicognani et al. (2008), this construct allows the well-being of individuals nested in social structures to interact, given that its dimensions cover the evaluation that individuals make of their overall performance in society. The authors pointed out that social context evaluation is carried out through social integration and social contribution. The evaluation of other people is carried out through the dimension of social acceptance, and the evaluation of personal performance in society is carried out through the dimensions of social coherence and social actualization.

For children and adolescents, K-12 schooling is usually the first space for socialization and learning of social interaction outside the family (Oyarzún et al., 2017). In schools, the relational patterns learned in neighborhood contexts and cultural groups tend to be applied and reproduced (Ahmadi et al., 2020). The possibility of a positive school experience, then, is inscribed in a healthy ecological environment that favors a comprehensive curriculum for the development of all types of skills, including socioemotional learning, in the framework of a school culture that cares about the quality of life of its community, both individually and collectively (Benbenishty and Astor, 2005; Shirley et al., 2020).

However, research that considered well-being at school has focused mainly on indicators of subjective well-being indicators. Satisfaction with school (Oyarzún et al., 2017) is an evaluative judgment about school quality from the students’ perspective. There is far less research that addressed the social dimension of well-being and its relationship to the school. Recent research on well-being in the school context shows that it is not only about schools caring for and catering to the development and well-being of their students but also about students feeling that they belong to and feel attached to their schools and students perceiving the necessary agency to assume that they can make significant contributions to their schools (Khoury-Kassabri et al., 2004). In this sense, the literature on SWS can be closely related to the literature on school belongingness (Boston and Warren, 2017; Ahmadi et al., 2020), in the sense that both constructs posit the need for students to feel that they belong to and feel engaged with their schools. However, the construct of social well-being highlights the need to provide opportunities that may make students feel not only engaged but also a significant part of and contributor to their schools, highlighting the role of students’ agency and participation. For example, Cicognani et al.’s(2001,2008) studies have shown that the social well-being of students increases based on participation and a sense of belonging to the educational community. In Berkman et al.’s (2000) study, socially oriented behaviors and feeling of belonging to a significant social context (sense of community) increased students’ social well-being and reinforced their social participation.

From a socioecological approach (Bronfenbrenner and Morris, 1998; Benbenishty and Astor, 2005; Astor and Benbenishty, 2018), social well-being can be interpreted as the result of the interaction of the individual with different social environments (Shirley et al., 2020). Thus, a student’s personal characteristics, when interacting with their family and school context, will influence the quality of well-being. According to Bronfenbrenner and Morris (1998), the factors that influence a person’s experience are located at different levels, where family and school as microsystems offer opportunities to develop belongingness to different significant groups. The development of close social relationships enhances skills to understand and interact in the social world (Shirley et al., 2020). Therefore, the study of SWS requires considering the different microlevels in which students develop, especially their family and school contexts.

In the next pages, we provide evidence of factors associated with SWS. Although some of the literature reviewed dealt specifically with social well-being in the school context, most reports focused on social well-being in general or subjective well-being in the school context.

Regarding individual factors, results concerning the influence of age and gender on social well-being have not been conclusive. Some studies showed significant differences between age and satisfaction with life. For example, Chen et al. (2019) found that boys and girls obtained higher scores than adolescents. Similarly, Petito and Cummins (2000) found that well-being decreased with age, and other studies have confirmed that as adolescence progresses, satisfaction with life decreases (Suldo and Huebner, 2004; Tomyn and Cummins, 2011; Casas et al., 2012; Oyanedel et al., 2015). Gender differences also seem to be present during adolescence. For example, Venning et al. (2013), in a representative sample of South Australia adolescents, found significant gender differences, whereby boys in metropolitan areas with better well-being were more likely to engage in healthy behaviors such as sleeping longer than their counterparts in rural areas. Cicognani et al. (2008), in a study carried out with a sample of American, Italian, and Iranian university students, found no differences by age but did find gender differences, particularly between American or Italian students and Iranian students.

These findings suggest that the gender variable has a cultural correlate that affects social well-being. An extensive bibliographic review of the relationship among violence, discomfort, and gender showed that in underdeveloped cultures, particularly sub-Saharan Africa, women present lower levels of well-being (Dunne et al., 2006). Le Mat (2016), in an investigation with female Ethiopian students, showed that patriarchal structures were associated with lower well-being among women. Regarding the relationship between the specific dimensions of Keyes’s (2003) social well-being scale and gender, Zubieta et al. (2012) found greater autonomy relative to social pressure among men but a better perception of their social contribution and ability to develop their capacities in the social context among women. Zubieta and Delfino (2010), in a study with university students from Buenos Aires, found that women had better social relationships and felt more useful than men. However, these studies found gender differences among adults. To our knowledge, no studies have reported gender differences in SWS in school-aged children and adolescents.

Research addressing the associations between children’s and adolescents’ socioeconomic status (SES) and social well-being has not been entirely conclusive. For the most part, the literature supports a positive and significant relationship between SES and social well-being. In Cicognani et al.’s (2008) study, SES was related to a sense of belonging to the school. A possible explanation is that people with higher SES have more significant and better opportunities to participate in social life. These findings are consistent with previous studies that indicated that students with low SES show little connection and sense of belonging to their school, which affects their social well-being (OECD, 2013; Chiu et al., 2016). Sarriera et al. (2015) investigated the relationship between the perception of material resources (clothes in good condition to go to school, access to a computer at home, and access to the internet and a mobile phone) and satisfaction with life in a sample of 953 boys and girls between 10 and 14 years old in eight countries. The study showed a positive and significant relationship between material resources and life satisfaction. This relationship was higher in countries with less access to these material resources (Algeria, Uganda, and South Africa) and lower in countries with better access conditions (South Korea, England, and Spain). In Chile, research has shown that lower SES is related to lower happiness, using a single-item measure of happiness (Oyanedel et al., 2015). In contrast, Kwan (2008) found that life satisfaction among adolescents from the same geographical context is independent of their economic situation.

Other factors act on an individual level but are related to students’ academic and social experiences in their school. The sense of belonging to a school is positively associated with attendance (Rosenfeld et al., 2000), greater motivation and effort (Sánchez et al., 2005), and academic performance and success (Barber and Olsen, 1997; Blum, 2005; Boston and Warren, 2017; Korpershoek et al., 2019), measured as grade point average (GPA) (Roeser et al., 1996), and completion rates at school (Connell et al., 1995). However, we found no studies relating these variables to SWS. Therefore, there is a need to understand if and how academic performance, as measured by students’ GPA, contributes to their SWS. Likewise, the quality and character of students’ social interactions with their peers in school might affect their SWS. In this respect, we know that social support is a relevant characteristic for the construction of satisfaction with life and students’ individual and social well-being (Benson and Scales, 2009; Rodríguez et al., 2016). Peer social support has been found to moderate the levels of victimization and aggression among peers (Villalobos et al., 2016), helping students adapt to the school context. Relationship with peers seems to play a key role during adolescence, a stage in which students configure their identity based on their social network (San Martín and Barra, 2013). Along the same lines, several studies have shown that students who perceive their family relationships positively present less victimization by peers and teachers, particularly during adolescence (Demaray and Malecki, 2002; Moreno et al., 2009; Bokhorst et al., 2010).

In the school context, peer social support favors the perception of satisfaction with life, higher levels of self-esteem and subjective well-being, and a peaceful and non-violent attitude among peers (Clara et al., 2003; Ben-Ari and Gil, 2004; Zhang and Zhang, 2012). Positive associations have been found between students perceiving that it is possible to establish friendships in school and their social well-being (Chu et al., 2010). However, peer relationships are not the only type of relationship that seems to influence students’ SWS. Their relative contribution regarding other important relationships, such as with their family and teachers, has been scarcely studied and needs to be considered jointly. In this sense, Gutiérrez et al.’s (2017) findings that academic social support from peers, when considered together with social support from teachers and family, was not associated with students’ satisfaction with school is consistent with Chu et al.’s (2010) meta-analytic findings that social support received from peers weakly predicted social well-being.

Family characteristics might also make a relevant contribution to students’ SWS. Research on subjective well-being has shown that families act as a moderating variable of subjective well-being (González et al., 2015; Gutiérrez et al., 2017). Likewise, family is relevant for the construction of a sense of social belonging, especially among adolescents (Oliva et al., 2002; Bernal et al., 2013). Satisfaction with family has been shown to influence adolescents’ development and emotional stability (Olson, 1985). Establishing positive emotional ties with the family system contributes to maintaining not only intrafamily ties but also those established with others (Crespo, 2011; Bernal et al., 2013). Living with both parents is related to higher levels of well-being, mainly because it’s positive relationship with family income (Berger and McLanahan, 2015); and parent involvement and time spent with parents also contribute significantly to children’s well-being (Moreno and Vicente, 2019). Studies have shown that when the family climate is positive, life satisfaction increases in adolescents, directly influencing self-esteem and decreasing the depressive symptoms they may experience (Jiménez et al., 2008; Tercero et al., 2013; Organización Mundial de la Salud, 2018). In addition, its impact is significant in the construction of the self-image that students develop in school. Gutiérrez et al. (2017) found that perceptions of both school and family climate contribute to students’ satisfaction with life. Platsidou and Tsirogiannidou (2016) found similar evidence, adding that family climate was especially relevant for satisfaction with life among students who reported higher levels of family cohesion and communication.

Last, student turnover is a relevant issue to consider regarding students’ social well-being. Continuous academic trajectories not only contribute to better academic results (Riglin et al., 2013) but also provide opportunities for a social experience based on friendships and future projections (Rodríguez et al., 2016; Villalobos et al., 2016). In contrast, frequent changes of schools can signify an anxious experience that affects the mental health of students (Ng-Knight et al., 2019). Now, the impact of vital changes on well-being is not the same for all people (Luhmann et al., 2012; Kettlewell et al., 2020). In the case of children and adolescents, they face different changes and transitions, at both developmental and social levels, which undoubtedly affect well-being and can alter life trajectories (Benner et al., 2017). School changes are no exception, because they imply making a significant adjustment at social and academic levels and can cause vulnerability at a psychological level as a risk factor for mental health and well-being (Slee and Allan, 2019). Changing schools reconfigures a student’s social network (Benner et al., 2018), and very few students maintain their friendships after an institutional change (Ng-Knight et al., 2019).

Although in some cultures changing schools is a rare phenomenon and, therefore, might be considered an individual characteristic of certain students who, due to various difficulties in their current school, ask to be or are transferred to a different school, in Chile, changing schools is a highly frequent situation for students. According to Treviño et al.’s (2016) national cohort study, only 14% of senior high school students finish their senior year with the same class they started in their freshman year. Although most of this is due to changes in classroom (51%), 20% is due to changes of school. The market-driven school choice model implemented in Chile for nearly four decades has an impact on frequent changes of schools (Plank and Sykes, 2003; Forsey et al., 2008; Musset, 2012). Historically, Chilean families have had the freedom to choose a school, with no controlled or regulated strategies. Families select their child’s school in the public or private sector, regardless of their place of residence, with no limit on the number of changes (Hernández and Raczynski, 2015). Therefore, in a market-driven educational system, the families decide in which school their children will study, regardless of the proximity of the school to the neighborhood where the student lives. Families can move their children freely from one educational establishment to another, in search of a better-quality education (Zamora and Moforte, 2013). In general, the decision to change schools reflects the satisfaction of the parents with the educational service provided, which does not always correspond with actual student achievement scores but on factors such as trust in the school (Zamora, 2011). Student turnover due to changing schools affects educational outcomes, even more so for the most vulnerable students, who are less able to mitigate the effects of the change in learning pace (Bonal and Zancajo, 2020). Therefore, in this study, we viewed student turnover as a family characteristic.

Finally, regarding school factors, a positive school climate undoubtedly contributes to an experience of individual and social well-being (Hamre and Pianta, 2001; Blum, 2005; Lester and Cross, 2015; Cruz et al., 2020). A positive school climate includes fair and known rules, high teacher expectations, student participation, and teacher support (Berkowitz et al., 2017). Also, school connectedness and positive student–teacher relationships have been shown to have a significant relationship to school satisfaction in middle and high school students (Zullig et al., 2011). Clear, well-known, and fair norms and fair treatment are factors that enhance school climate (Molinari and Mameli, 2018). A school experience perceived as unfair can negatively affect students’ identity, sense of coherence, and performance (Ascorra et al., 2014). Some research linked fair norms to SWS. Fair, clear, and well-known rules and fair play have been identified as variables that favor social well-being and affect academic performance (Cobo et al., 2020). Along the same lines, Gage and Berliner (1996) suggested that injustices and the lack of fair norms negatively affect students’ sense of coherence and performance. Positive relationships between teachers and students, as well as support from teachers to students, play a determining role in the quality of children’s experience in school (Hamre and Pianta, 2001). Teacher support is one of the strongest predictors of school belongingness (Allen et al., 2018) and students’ well-being (Chu et al., 2010). The support of teachers and other adults who encourage participation in school improves school climate and constitutes a protective barrier against students’ risky behaviors (McNeely and Falci, 2004; Drolet et al., 2013). Conversely, school disengagement relates to the rupture of membership in social networks and the loss of security in local contexts (Alonso-Martinera, 2017). As a consequence, a school in which its members feel unengaged may attain low levels of academic performance, low levels of subjective well-being among its members, a more violent school climate, and a higher level of perception of subjective uneasiness (Benbenishty and Astor, 2005; Bilbao et al., 2014; Wang and Degol, 2016).

Research is also consistent in pointing out a negative relationship between well-being and peer victimization. The longitudinal study by Looze et al. (2020) of a sample of 232 Australian teenagers concluded that the emotional well-being of both boys and girls declined between 2009 and 2013. Perceived pressure of schoolwork was associated with this decline. Improved communication between parents and adolescents and decreased bullying victimization explained why emotional well-being remained stable between 2013 and 2017, despite a further increase in schoolwork pressure. Lester and Cross (2014) found that men who had been victimized in school had worse well-being and presented behavioral and hyperactivity problems, whereas women who had been victimized by peers also had decreased well-being, presenting emotional symptoms.

School factors are related to students’ experiences with their teachers. On the one hand, research has suggested that the victimization of students by teachers is related to the victimization of teachers by students. This spiral of aggression is known as cross victimization (Khoury-Kassabri, 2006; López et al., 2020). The explanatory hypothesis of this spiral of violence is related to teachers’ beliefs that through their own aggression, they might stop the aggression of students or achieve the academic objectives that the school has demanded of them (Innes and Kitto, 1989; Emmer and Hickman, 1991). As a result of this interaction, teachers feel overwhelmed and begin to perceive the school as a threatening environment, deteriorating the school climate and social well-being (Martin et al., 1999). Victimization by teachers and other school staff members has detrimental effects on the psychological and social well-being of children, which leads to feelings of sadness and lower self-esteem (Pottinger and Stair, 2009). In addition, these children show less motivation for learning and have lower academic achievements (Pinheiro, 2006). The violence of an adult toward a child is rooted in power relations and beliefs linked to age and gender regarding legitimate ways of instilling discipline. International research suggested that the victimization of teachers toward students is associated with cultural values. These values are the basis of violence against students. Because it follows cultural patterns, this type of violence is usually invisible. The impact of cultural values is especially relevant for female students from developing countries victimized by male teachers (Le Mat, 2016). On the other hand, students’ perception of their teachers’ well-being also has an impact on students’ social well-being. According to Shirley et al. (2020), well-being should be understood at the community level and not in individual terms. Following socioecological theory (Bronfenbrenner, 1992; Astor and Benbenishty, 2018), if members of the educational community suffer, including teachers, students will see their well-being decrease (Harding et al., 2019).

In Chile, research on social well-being has been scarce compared with evidence reported on subjective well-being and its measurement and contributions (Oyanedel et al., 2015; Oyarzún, 2018). Several qualitative studies on subjective well-being have highlighted the need to consider variables of interpersonal relationships and the material and affective environments in which students engage and participate, to quantify the school experience in a positive and collective way (Berger et al., 2009; Fernández et al., 2020; Ramírez et al., 2021). A few quantitative studies positioned social well-being together with school climate and teacher support as a moderating variable to explain the association between academic results and the subjective well-being of students (Bilbao et al., 2014) and to analyze the processes of acculturation of minority students (Mera et al., 2017; Céspedes et al., 2019).

However, none of these studies systematically investigated whether, which, and to what extent–when considered relative to their joint contribution–individual, family, and school characteristics are associated with better levels of SWS. The framework of SWS posits that the context of schools can shape students’ perception of feeling integrated and making significant contributions to school. However, not much is known regarding the joint contribution of individual, family, and school characteristics to students’ SWS. Furthermore, from a socioecological perspective (Bronfenbrenner, 1992; Astor and Benbenishty, 2018), SWS can be understood as being shaped by interacting contexts that are constantly evolving. Outside contexts, such as student characteristics (i.e., gender) and family demographics (i.e., poverty) and characteristics, may influence students’ experiences in the school, but they do not predetermine what happens in school nor SWS. This is because the school’s internal context moderates these external influences and helps shape students’ experiences, perceptions, emotions, and behaviors. Therefore, and considering that students are nested in schools, we hypothesized that school-level factors, both when reported individually by students and considering the school mean, would have a higher contribution than individual and family factors to SWS. Hence, this study analyzed, through a multilevel design, the joint contribution of individual, family, and school characteristics to students’ SWS.

## Materials and Methods

### Participants

This study used a probabilistic, stratified, and two-stage sample (region and school) of students in traditional schools in urban zones of the 16 regions of Chile. The sampling framework used was the 2017 National School Enrollment Registry from the Chilean Ministry of Education. Later, and to include the school’s SES in the data, the National School Vulnerability Index was linked to the original database.

The sample consisted of 6,389 students (56% female) enrolled in fifth to 11th grades from 212 schools. The sample size had an observed error of ±1.2, assuming a maximum variance, a 95% confidence level, and a response rate of 88.9%. Sample age ranged from 10 to 20 years (*M* = 13.89, *SD* = 2.08). Students were enrolled in all types of officially recognized schools: public subsidized schools (48.7%), public schools (37.5%), private schools (7.9%), and another administrative dependency (5.9%). According to the Chilean Index of School SES (known as school vulnerability), 49.4% of school catered to students with low SES, 28.2% to medium SES, and 22.4% to high SES.

### Measures

#### Criterion Variables

##### Social well-being at school

Bilbao et al. (in press) applied Keyes’ (1998) construct of social well-being to the school context and developed measures of SWS reported by students and teachers. SWS was defined as students’ and teachers’ evaluation of the circumstances and functioning of their school. In this study, we used Bilbao et al.’s (2014) adapted instrument of SWS, reported by students. This instrument consisted of an adapted form of the Social Well-Being Scale developed by Keyes (1998) and later modified by Blanco and Díaz (2005) to assess five dimensions of social well-being. The adaptation consisted of replacing the word “society” for “school,” as a way to contextualize students’ evaluation of their society through a more proximal, context-based experience of school as society. These changes were first piloted qualitatively with students to see if they made sense and if the items were comprehensible. After that, psychometric properties were studied, with adequate results. These two processes produced 21 suitable items, from the 25 proposed by Blanco and Díaz (2005). The adapted version of 21 items had a five-point Likert scale (from 1 = *I completely disagree* to 5 = *I completely agree*) and measured five dimensions of social integration (four items), social acceptance (six items), social contribution (two items), social actualization (four items), and social coherence (five items), but with a different phrasing. Instead of “in this society,” the item was modified to “in this school.” The internal consistency for the full scale was 0.808. We used a standardized index of SWS from the average of item responses. Confirmatory factor analyses (CFAs) showed suitable fit [χ^2^_(__181__)_ = 3,643.61, *p* < 0.001; CFI = 0.952; root mean square error of approximation (RMSEA) = 0.041; standardized root mean square residual (SRMR) = 0.041].

#### Predictors Related to Students’ Individual Characteristics

##### Gender and age

These variables were measured as reported by students.

##### Number of information and communication technologies at home

Due to the absence of questions related to family income and parents’ schooling level, we used the number of information and communication technologies (ICTs) as a proxy variable of students’ family wealth and SES (Buchmann, 2002). This index was calculated as the sum of five items n.

##### Students’ grade point average

We used the students’ GPA at the end of the school year. This was a continuous variable, which in Chile is measured on a scale from 1 to 7, where a higher score means a better GPA.

##### Satisfaction with peer friends

Three questions asked about how satisfied students were with their relationships with peers and friends (e.g., “How satisfied are you with your friends?”). One of these questions was measured on an 11-point Likert scale, from 0 = *not satisfied at all* to 10 = *completely satisfied*, and two were measured on a four-point scale. We used a standardized index from the average of responses to these three items. CFA showed suitable fit (χ^2^_(__2__)_ = 41.83, *p* < 0.001; CFI = 0.977; RMSEA = 0.051; SRMR = 0.023).

#### Predictors Related to Family Characteristics

##### Family satisfaction

We used an adapted form of the Family Satisfaction Scale developed by Olson and Wilson (1982). This scale is composed of 10 items that measure cohesion, adaptability, and communication in family dynamics measured with an 11-point Likert scale (from 0 = *do not agree at all* to 10 = *totally agree*, α = 0.926 for this sample). We used a standardized index from the average of responses to the items. CFA showed suitable fit (χ^2^_(__35__)_ = 2,058.57, *p* < 0.001; CFI = 0.960; RMSEA = 0.088; SRMR = 0.033).

##### Change of schools

The students were asked how many times during the last 3 years they had changed schools. This single question was measured on a four-point Likert scale, from 0 = *never* to 3 = *three or more times*.

#### Predictors Related to School Characteristics

##### School climate

We used a short version of Benbenishty and Astor’s (2005) School Climate Scale, as adapted by López et al. (2014). The scale is composed of 10 items with a four-point Likert scale (from 1 = *I completely disagree* to 4 = *I completely agree*). The scale assesses teacher social support (four items, α = 0.839), fair norms (three items, α = 0.762), and students’ participation in school (three items, α = 0.785). We created an index using the average of item responses for every dimension and the full scale. CFA showed suitable fit [χ^2^_(__81__)_ = 2,186.70, *p* < 0.001; CFI = 0.986; RMSEA = 0.052; weighted root mean square residual (WRMR) = 2.56]. We calculated the school average of the full index.

##### Peer victimization

We used the School Victimization Scale developed by Furlong et al. (1991), modified by Benbenishty and Astor (2005), and later adapted to the Chilean context by López et al. (2014). This scale assesses the prevalence of being victimized by school peers in the last month. It features 18 items aggregated in the following five dimensions: threats (four items), physical victimization (four items), sexual victimization (three items), and verbal victimization (seven items). We created an index using the average of item responses for every dimension and the full scale. CFA showed suitable fit (χ^2^_(__131__)_ = 8,174.13, *p* < 0.001; CFI = 0.907; RMSEA = 0.090; SRMR = 0.059). At the school level, we calculated the school average of the full index.

##### School socioeconomic status

A categorical variable measuring school SES, developed by the Chilean National Board of Assistance and School Scholarships (Junta Nacional de Auxilio Escolar y Becas), was included. This index classifies schools according to the percentage of enrolled students with vulnerability conditions, which is understood as low SES (range index = 0–100). Three categories were created, with high scores meaning high vulnerability, that is, low SES: (a) low vulnerability, if the vulnerability index is between 0 and 56 points; (b) medium vulnerability, if the vulnerability index is between 57 and 72 points; and (c) high vulnerability, if the vulnerability index is between 73 and 100 points.

##### Students’ perception of teachers’ well-being

This scale was created *ad hoc* in a previous study (Bilbao et al., 2014) and measures students’ perceptions about their teachers’ well-being (e.g., “My teachers are happy in this school”) with seven items on a five-point Likert scale (from 1 = *completely disagree* to 5 = *completely agree*). We created an index using the average of item responses for the full scale. At the school level, we calculated the school average of the full index. CFA showed suitable fit (χ^2^_(__2__)_ = 173.27, *p* < 0.001; CFI = 0.988; RMSEA = 0.107; SRMR = 0.017).

##### Teacher-to-student victimization

Benbenishty and Astor’s (2005) victimization scale, adapted by López et al. (2014), measures the prevalence of teacher-to-student victimization in the last month (e.g., “A teacher mocked, insulted or humiliated you”) with four items (α = 0.859 in this sample). We created an index using the average of item responses for the full scale. CFA showed suitable fit (χ^2^_(__181__)_ = 3,643.61, *p* < 0.001; CFI = 0.952; RMSEA = 0.041; SRMR = 0.041). At the school level, we calculated the school average of the full index.

### Analytic Plan

We first conducted descriptive analyses of the study variables and calculated the bivariate associations of all predictor variables with reports of SWS. Later, we performed a two-level linear multilevel analysis on the criterion variable to test the effects of predictors related to students, their families, and their schools at individual (level 1) and school (level 2) levels. The individual-related demographic variables were entered in the first model. In the second model, we added the family-related variables. In the third model, we introduced students’ individual experiences in school through GPA, peer victimization, and school climate and their perceptions of teacher-related variables. In the final model, we included the school-related variables, measured as the school average and school SES.

### Ethics Statement

This study was carried out in accordance with the recommendations of the National Agency of Science and Technology of Chile with written informed consent from all subjects. All subjects gave written informed consent in accordance with the Declaration of Helsinki. The protocol was approved by the Ethics Committee of Pontificia Universidad Católica de Valparaíso.

## Results

### Description of Study Variables and Association Between Social Well-Being at School and Study Variables

Table 1 shows the descriptive statistics of the study variables and the bivariate correlations between the predictors and reports of SWS. All variables showed a significant correlation with SWS, except for the number of ICTs. Age, number of school changes, peer victimization dimensions, and teacher-to-student victimization had negative correlations with SWS.

**TABLE 1:** Descriptive statistics and bivariate correlations at student level of study variables with social well-being at school.

Variable	Mean	SD	(1)
(1) Social well-being at school	0.01	0.58	1
Gender (female = 1)	0.56	0.50	0.05*
Age	13.89	2.08	−0.17*
Number of ICTs	9.80	3.95	−0.02
Family satisfaction	7.58	2.13	0.29*
School changes	0.81	0.96	−0.08*
2018 GPA	5.63	0.85	0.18*
Satisfaction with peers and friends	0.01	0.72	0.48*
School climate: teacher social support	2.99	0.74	0.53*
School climate: clear norms	3.06	0.72	0.49*
School climate: student participation	2.99	0.70	0.48*
Peer victimization: threats	0.39	0.97	−0.24*
Peer victimization: physical	0.55	1.09	−0.22*
Peer victimization: sexual	0.26	0.71	−0.20*
Peer victimization: verbal	1.60	2.02	−0.27*
Perceived teachers’ well-being	3.85	0.85	0.50*
Teacher-to-student victimization	0.30	0.82	−0.23*

ICTs, information and communication technologies; GPA, grade point average.

*p < 0.001.

### Multilevel Regression Analysis Predicting Social Well-Being at School

The results of the multilevel regression analysis of students’ reports of SWS are shown in Table 2. In the first model, featuring individual-related variables, findings show that being female was associated with higher social well-being (*b* = 0.05, *p* < 0.01) and that age (*b* = −0.04, *p* < 0.001) and number of ICTs (*b* = −0.01, *p* < 0.01) predicted lower levels of well-being in school. In Model 2, the family-related variables were added to the previous model, with no changes in significance for gender, age, and ICT possessions. Higher family satisfaction was positively associated with SWS (*b* = 0.07, *p* < 0.001), and more changes of schools (*b* = −0.03, *p* < 0.01) predicted a lower level of SWS. Model 3 shows that when introducing the school-related variables, the number of changes of schools lost significance. Students’ individual GPA at the end of the school year (*b* = 0.05, *p* < 0.001) and their satisfaction with peers and friends (*b* = 0.13, *p* < 0.001) were both positively associated with SWS. All school climate dimensions were statistically significant and predicted higher SWS, with participation (*b* = 0.08, *p* < 0.001) and teacher social support (*b* = 0.14, *p* < 0.001), showing the highest effect on SWS. The threat (*b* = −0.03, *p* < 0.01) and verbal (*b* = −0.01, *p* < 0.001) dimensions of peer victimization were negatively and statistically significant predictors of SWS. Students’ perception of teachers’ well-being in school (*b* = 0.14, *p* < 0.001) predicted a higher level of SWS, and teacher-to-student victimization (*b* = −0.03, *p* < 0.001) had a negative effect on SWS. Model 4 introduced the school-related variables at level 2. At the student level, none of the variables lost significance compared with the previous model. At the school level, only school climate was statistically significant and predicted higher SWS (*b* = 0.15, *p* < 0.001). The variables related to school SES, teachers’ well-being, and teacher-to-student victimization at the school level were not statistically significant.

**TABLE 2:** Summary of multilevel linear regression analysis for variables predicting social well-being at school at individual and school levels (*N* = 6,389 students at 212 schools).

	Null model		Model 1		Model 2		Model 3		Model 4	
Variables	*b*	(SE)	*b*	(SE)	*b*	(SE)	*b*	(SE)	*b*	(SE)
Constant	0.01 (0.02)	(0.02)	0.56*** (0.09)	(0.09)	−0.09 (0.09)	(0.09)	−1.47*** (0.09)	(0.09)	−1.83*** (0.19)	(0.19)
**Student level**										
Gender (female = 1)			0.05**	(0.02)	0.06***	(0.02)	0.04***	(0.01)	0.04**	(0.01)
Age			−0.04***	(0.01)	−0.03***	(0.01)	−0.01***	(0.00)	−0.01**	(0.00)
Number of ICTs			−0.01**	(0.00)	−0.01***	(0.00)	−0.01***	(0.00)	−0.01***	(0.00)
Family satisfaction					0.07***	(0.00)	0.01***	(0.00)	0.01***	(0.00)
Change of schools					−0.03**	(0.01)	−0.01	(0.01)	−0.01	(0.01)
2018 GPA							0.05***	(0.01)	0.05***	(0.01)
Friends satisfaction							0.13***	(0.01)	0.13***	(0.01)
School climate: teacher social support							0.14***	(0.01)	0.14***	(0.01)
School climate: fair norms							0.06***	(0.01)	0.06***	(0.01)
School climate: student participation							0.08***	(0.01)	0.08***	(0.01)
Peer victimization: threats							−0.03**	(0.01)	−0.03**	(0.01)
Peer victimization: physical							0.00	(0.01)	0.00	(0.01)
Peer victimization: sexual							−0.00	(0.01)	−0.00	(0.01)
Peer victimization: verbal							−0.01***	(0.00)	−0.01***	(0.00)
Perceived teachers’ well-being							0.14***	(0.01)	0.14***	(0.01)
Teacher-to-student victimization							−0.03***	(0.01)	−0.03***	(0.01)
**School level**										
**School vulnerability index (reference: low-vulnerability school)**										
Medium-vulnerability school									−0.01	(0.02)
High-vulnerability school									−0.01	(0.02)
School climate: total (school average)									0.14*	(0.06)
Peer victimization: total (school average)									−0.01	(0.01)
Perceived teachers’ well-being (school average)									−0.01	(0.04)
Teacher-to-student victimization (school average)									−0.01	(0.04)
Variance components	Null Model		Model 1		Model 2		Model 4		Model 6	
Student-level variance	0.297	(0.007)	0.295	(0.007)	0.271	(0.006)	0.180	(0.005)	0.180	(0.005)
School-level variance	0.034	(0.005)	0.025	(0.004)	0.022	(0.003)	0.005	(0.001)	0.004	(0.001)
% Level 1 variance explained	Base		0.87%		8.74%		39.63%		39.49%	
% Level 2 variance explained	Base		24.92%		35.56%		83.77%		88.88%	

*Standardized coefficients reported. Standard errors in parentheses. Explained variance compared with null model.*

ICTs, information and communication technologies; GPA, grade point average.

*p < 0.05, **p < 0.01, and ***p < 0.001.

The bottom part of Table 2 shows the partitions of the variance components of the criterion variable at the student and school levels and the explained variance compared with the null model of the specifications. The differences in SWS were concentrated at the individual level. However, even though variation at the school level was relatively low compared with the individual level, the proportion of the explained variance shows that the models explained more of the variance at the school level. Given the low variance, including variables at both levels increased the percentage of explained variance.

Robustness checks were conducted using the dimensions of the SWS scale as the criterion variable: social integration and contribution, social acceptance, social actualization, and social coherence. Results of these models predicted similar results as observed using the whole SWS scale. Major differences were (a) the significant coefficient of school-level peer victimization predicted lower scores of social acceptance and social coherence, and (b) only the social actualization dimension replicated the significant coefficient of school climate at the school level that was present in the SWS scale. Results of these estimations are shown in Supplementary Tables.

## Discussion

This study analyzed, through a multilevel design, the joint contribution of individual, family, and school characteristics to students’ SWS. Taking a socioecological approach (Benbenishty and Astor, 2005; Astor and Benbenishty, 2018), which places the school at the center as mainly responsible for students’ SWS, we hypothesized that school-level factors, both when reported individually by students and when considering the school mean, would have a higher contribution than individual and family factors to SWS. Overall, the findings of this study provide evidence in favor of this hypothesis. These are highly relevant findings considering that adequate school policies, reforms, and interventions can change how schools cater to the social, emotional, and academic needs of all students (Astor and Benbenishty, 2018) by promoting and facilitating school well-being (Keyes, 2009) and that schools are more permeable to interventions than individual and family characteristics.

First, findings show that concerning individual demographic variables, being female was associated with higher SWS. These findings are in line with findings from other studies that show that girls have higher levels of well-being in school (Cicognani et al., 2008; Le Mat, 2016). A hypothesis explaining this gender difference in Latin America is the change in women’s position in the sociopolitical area. In recent years and as a result of massive feminist mobilizations, women have begun to have a more significant impact in the social sphere (Zubieta and Delfino, 2010).

Evidence has shown that well-being decreases as age increases (Petito and Cummins, 2000; Huebner et al., 2004; Suldo and Huebner, 2004; Tomyn and Cummins, 2011; Casas et al., 2013). Adolescents begin to question or analyze social structures, which are discovered to be unfair and unequal. As in previous studies, our findings show that age was negatively associated with SWS, meaning that younger students reported higher SWS. Recent explanations for these findings include that whereas children develop a greater sense of belonging to school because it is a meaningful space to participate in and contribute to the social world and explore their own interests, in contrast, adolescents find their well-being in other reference groups (Petito and Cummins, 2000; Zubieta et al., 2012; Oyarzún and Loaiza, 2020).

Findings regarding SES are not consistent with the literature. Results show that in all models, the number of ICT possessions in the household reported by students, used as a proxy variable of SES, was negatively associated with SWS, although the coefficients were relatively low compared with other predictors. Vaz et al. (2015) found in a sample of 12-year-old students in the final year of study of primary school in Australia that household SES did not have effects on school belongingness, arguing that students’ social standing could not be relevant in this age group. At the school level, the school vulnerability index presented no variability associated with the students’ perception of SWS, showing that a higher proportion of students with lower SES did not have a significant association with SWS. Therefore, analysis of the relationship between the number of ICTs and SWS offered inconclusive evidence and should be further explored.

Second, family-related factors were significantly associated with SWS in the expected direction. Higher satisfaction with family was positively associated with SWS, and more school changes was negatively associated with SWS. These findings support those reported by Martínez Ferrer et al. (2011), who found that a positive perception of family climate was associated with higher well-being levels. Although school changes are an opportunity for new social interactions, they rarely allow students to maintain previous relationships, which is relevant for the construction of SWS (Riglin et al., 2013; Rodríguez et al., 2016). Given this, an experience of well-being in the previous school could cushion anxiety due to transfer or change of school (Kettlewell et al., 2020) and help students face future transfers or school changes. Therefore, schools should consider the negative impact of school changes on children’s well-being in initiatives that allow them to enter this new social world in ways that help them develop a sense of belonging and new meaningful relationships with others.

When the variables measuring students’ experiences at school were incorporated, the number of changes of school lost significance. This non-significance remained throughout the rest of the models, implying that the quality and character of students’ experience in schools carry more weight than the fact that for some students their permanence in school might be shorter than the rest of their peers. This is significant evidence, considering that in Chile, approximately 20% of students change schools throughout the course of high school (Treviño et al., 2016). The context explaining this high number is a market-driven educational model that drives parents to “choose the best school” for their children, implicitly encouraging parents to change their children’s schools constantly.

Students’ individual GPA at the end of the school year and their satisfaction with peers and friends were both positively associated to SWS. These findings are consistent with research that showed that positive relationships with peers and friends are relevant for the construction of well-being during childhood and adolescence (Benson and Scales, 2009; Rodríguez et al., 2016; Villalobos et al., 2016). However, their contribution to SWS slightly diminished when studied jointly with their perception of teachers’ well-being, which is consistent with Chu et al.’s (2010) meta-analysis findings, in the sense that teachers make significant contributions to students’ well-being.

As expected, students’ perception of their teachers’ well-being in school predicted a higher level of SWS. These findings are consistent with socioecological approaches in the sense that the well-being of the student is permeable to the well-being of the teacher. These findings suggest that the school should be interpreted as a system, wherein its sustainability depends on the well-being of the entire community (Shirley et al., 2020). Considering well-being as a collective and not just an individual phenomenon (Alfaro et al., 2015; Harding et al., 2019; Ahmadi et al., 2020; Shirley et al., 2020) opens an important venue for fostering students’ well-being, through encouraging elements that contribute to teachers’ well-being. Surprisingly, there is a scarcity of research linking the evidence on teachers’ work and working conditions (Maldonado and Cornejo, 2020) to students’ school well-being and belongingness. In contrast, higher reports of teacher-to-student victimization were associated with lower reports of SWS. These findings provide evidence of the need to cater to a rights-based perspective regarding teacher–student relationships (Benbenishty et al., 2019).

Likewise, all school climate dimensions were statistically significant and predicted a higher report of SWS, with student participation and teacher social support showing the highest effect. Interestingly, the specific effect of verbal types of victimization, not physical or sexual victimization, made a difference for students’ SWS. Verbal victimization is known to be one of the most frequent forms of peer victimization in different cultures (López et al., 2018), but it tends to be overlooked by schools as a “natural” way in which students treat each other (Khoury-Kassabri, 2006). These findings provide evidence of the need to consider the effects and consequences of verbal peer victimization in students’ sense of belonging and contributing to their schools.

Overall, the strength of the coefficients for these school-related factors support our hypothesis that the school experience, beyond students’ individual sociodemographic and family characteristics, contributes more to their sense of feeling integrated, valuable, and contributing significantly to their school. Particularly relevant is the climate of the school. School climate has been defined as the quality and character of school life (Cohen, 2008). In this study, the dimensions of student participation and teachers’ social support proved to be particularly relevant. In the final model, school climate was the only school-related factor measured at the school level–that is, as the mean of students’ response in a given school–that contributed to explaining changes in students’ reports of SWS. This is very relevant, because demeaning behaviors related to peer victimization, particularly through threats and other verbal types of victimization, were significantly associated with SWS at the individual level but not the school level. These findings suggest that positive and engaging school climates may buffer the effect of unwanted peer behaviors on students’ SWS and of specific teachers’ possible demeaning behaviors toward some students.

In the final model, the demographic variables of age and gender lost their strength of contribution when incorporating family- and school-related factors. Likewise, family-related factors lost their strength of contribution (family satisfaction) and even lost significance (change of school) when incorporating school-related factors. In this final model, the highest contribution to SWS came from school-related factors. When considering students’ individual appraisals, the most important school-related factors were teachers’ social support, perception of teachers’ well-being, and satisfaction with peers and friends–in other words, teachers and friends. These findings suggest that academic achievement is significant, but not as important as teachers and friends, in shaping students’ sense of belonging and making an important contribution to their schools. When considering students’ mean reports at the school level, the only school variable that remained significant was school climate. In fact, the highest contribution of SWS was explained by the school-level aggregated perception of school climate. We argue that this is because the school’s internal context moderates external influences and helps shape students’ experiences, perceptions, emotions, and behaviors (Astor and Benbenishty, 2018).

These findings call for integrated policies and practices to foster students’ sense of belonging, feeling integrated, and contributing to their schools focused on school-level interventions to improve SWS through positive and engaging school climates that facilitate teachers’ well-being and foster students’ sense of agency. For school interventions and public policies, this is relevant because SWS may be considered an educational outcome, in the sense that schools and schooling should provide not only academic but also social development, which could be measured as schools’ responsibility to offer opportunities for social integration, acceptance, contribution, actualization, and coherence (Keyes, 2006). Within the current pandemic situation, these findings call on the need to provide sustainable opportunities to engage and maintain teachers’ and students’ SWS throughout the online, remote, and hybrid forms of education that are taking place throughout the world, as a way of preventing student disengagement and future dropout (Miranda-Zapata et al., 2018; Shirley et al., 2020).

Theoretically, this study makes a significant contribution to the field of positive mental health and social well-being among children and adolescents. Although studies on social well-being within these age groups are increasing, only a dozen of more than 2,000 studies published in Web of Science from 2004 to 2021 take on a social–ecological approach and include children and adolescents’ joint evaluation of their families, schools, and peers. This approach has an important theoretical contribution by helping comprehend the complexity of children and adolescents’ well-being, which is strongly determined by the school context and by significant others (Chu et al., 2010; Tomyn and Cummins, 2011; Alfaro et al., 2015; Ahmadi and Ahmadi, 2019; Ahmadi et al., 2020). The findings from this study allow a deeper theoretical comprehension of the specific role of each ecological frame that is involved in the social well-being of students. This is important given the fact that most studies on positive mental health have being related to satisfaction with life, affects, or psychological well-being, all variables at the individual level. The study of social well-being gives the opportunity to broaden the comprehension of adolescents’ global well-being and mental health, through understanding how they evaluate the quality of their relationships with others in social contexts that play a key role for them (Keyes, 2003, 2006, 2009, 2013). By taking into account the evaluation of the functioning and comprehension of the most relevant social context for children and adolescents–their schools–we provide a contextualized view of the central aspects of adolescents’ social well-being. Likewise, the adaptation of the original scale to the school context also is a contribution to the field, providing an adapted instrument that might be further used in future studies (see Supplementary Tables).

### Limitations and Future Directions

A limitation of this study was the cross-sectional design, given that it was not possible to identify causal relations between the study variables. Additionally, as a proxy of family SES, we used students’ reports of the number of ITC possessions. Even though it was used as a representation of the wealth of the household, it is not optimal because it did not consider other types of household possessions. There might also be inconsistencies between students’ and parents’ reports (Traynor and Raykov, 2013). Future studies should consider a longitudinal approach to help identify causal relationship between SWS and individual, family, and school-related variables. It is also necessary to include a more optimal measure of family SES. Future studies should explore the theoretical and empirical links between SWS and school belongingness and include families’ and teachers’ perception to complement students’ view in understanding the factors that contribute to SWS and how schools can foster students’ SWS.

## Data Availability Statement

The raw data supporting the conclusions of this article will be made available by the authors, without undue reservation.

## Ethics Statement

This study was carried out in accordance with the recommendations of the National Agency of Science and Technology of Chile with written informed consent from all subjects. All subjects gave written informed consent in accordance with the Declaration of Helsinki. The protocol was approved by the Ethics Committee of Pontificia Universidad Católica de Valparaíso.

## Author Contributions

VL: design of the study, proposal of theoretical framework, supervision of data analyses, and write-up of the manuscript. JT-V: co-design of the study, definition of measures and organization of data analysis plan, data analysis, and contributions to theoretical framework. PA: literature review, organization and write-up of the introduction and discussion. LG: co-participation in data analysis plan and execution and participation in discussion. SO: literature review, write-up of the introduction and discussion, and formatting and reference list. MB: proposal of the measure of social well-being at school and revision of the final manuscript. All authors contributed to the article and approved the submitted version.

## Conflict of Interest

The authors declare that the research was conducted in the absence of any commercial or financial relationships that could be construed as a potential conflict of interest.
