# Supplementary material for: Contributions of Individual, Family, and School Characteristics to Chilean Students’ Social Well-Being at School
Source: Front Psychol. 2021 Feb 26;12:620895. doi: 10.3389/fpsyg.2021.620895 (PMC7952761; doi:10.3389/fpsyg.2021.620895)
Supplement: Supplementary file 1 [file Data_Sheet_1.docx]

Social Well-Being at School Scale

Considering the last month at your school, please tell us how much you agree or disagree with the following statements:

[*En relación con el último mes en tu escuela, colegio o liceo, dinos qué tan de acuerdo o en desacuerdo estás con las siguientes afirmaciones:*]

**Social integration:**

1. I feel that I am an important part of this school's community. [*Siento que soy una parte importante de la comunidad de esta escuela*.]
2. I think the people at this school appreciate me as a person. [*Creo que la gente de esta escuela me valora como persona.*]
3. I feel close to other people in this school. [*Me siento cercano a otras personas de esta escuela.*]
4. This school is a source of well-being for me. [*Esta escuela es una fuente de bienestar para mí.*]

**Social contribution:**

1. I think I can contribute to this school. [*Creo que yo puedo aportar a esta escuela.*]
2. I think what I do is important to this school. [*Pienso que lo que hago es importante para esta escuela.*]

**Social acceptance:**

1. I think the people at this school are not trustworthy. [*Creo que la gente de esta escuela no es de confianza.*]
2. In this school, people think only of themselves. [*En esta escuela, las personas sólo piensan en sí mismas.*]
3. I think people in this school are selfish. [*Creo que la gente de esta escuela es egoísta.*]
4. People in this school are more and more dishonest. [*Las personas de esta escuela son cada vez más deshonestas.*]
5. In this school they do not care about other people's problems. [*En esta escuela no se preocupan de los problemas de otros.]*
6. I think people in this school should not be trusted. [*Creo que no se debe confiar en la gente de esta escuela.*]

**Social actualization**

1. I think the people at this school are kind. [*Creo que las personas de esta escuela son amables.*]
2. This school is always evolving. [*Esta escuela está en continuo desarrollo*.]
3. This school is a better place for us every day. [*Esta escuela es cada día un mejor lugar para nosotros.*]
4. This school does not offer opportunities for someone like me. [*Esta escuela no ofrece oportunidades para alguien como yo.*]

**Social coherence:**

1. I feel that this school is no longer moving towards something better. [*Siento que esta escuela ya no avanza hacia algo mejor.*]
2. I do not understand what is going on in this school. [*No entiendo lo que pasa en esta escuela*.]
3. This school is too complicated for me. [*Esta escuela es demasiado compleja para mí.*]
4. It is not worth the effort to understand this school. [*No vale la pena esforzarse en entender esta escuela.*]
5. There are so many different groups here that I cannot understand them. [*Acá hay muchos grupos tan diferentes que no puedo comprenderlos*.]
