# Supplementary material for: Contributions of Individual, Family, and School Characteristics to Chilean Students’ Social Well-Being at School
Source: Front Psychol. 2021 Feb 26;12:620895. doi: 10.3389/fpsyg.2021.620895 (PMC7952761; doi:10.3389/fpsyg.2021.620895)
Supplement: Supplementary file 2 [file Table_1.DOCX]

Supplementary Material

# Supplementary Table 1.

*Summary of multi-level linear regression analysis for variables predicting social integration and contribution at school at individual and school level (N=6,389 students at 212 schools)*

|  | Null Model | | Model 1 | | Model 2 | | Model 3 | | Model 4 | |
| --- | --- | --- | --- | --- | --- | --- | --- | --- | --- | --- |
| Variables | *b* | (SE) | *b* | (SE) | *b* | (SE) | *b* | (SE) | *b* | (SE) |
| Constant | 0.03 | (0.02) | 0.85*** | (0.11) | -0.43*** | (0.12) | -2.25*** | (0.11) | -2.70*** | (0.23) |
| *Student-level* |  |  |  |  |  |  |  |  |  |  |
| Gender (Female=1) |  |  | -0.03 | (0.02) | 0.01 | (0.02) | -0.01 | (0.01) | -0.01 | (0.01) |
| Age |  |  | -0.07*** | (0.01) | -0.05*** | (0.01) | -0.02*** | (0.00) | -0.02*** | (0.00) |
| Number of ICTs |  |  | 0.01** | (0.00) | 0.01* | (0.00) | 0.00 | (0.00) | 0.00 | (0.00) |
| Family satisfaction |  |  |  |  | 0.14*** | (0.01) | 0.04*** | (0.00) | 0.04*** | (0.00) |
| Change of schools |  |  |  |  | -0.00 | (0.01) | -0.00 | (0.01) | 0.00 | (0.01) |
| 2018 GPA |  |  |  |  |  |  | 0.03** | (0.01) | 0.03** | (0.01) |
| Friends satisfaction |  |  |  |  |  |  | 0.28*** | (0.02) | 0.28*** | (0.01) |
| School climate - Teachers social support |  |  |  |  |  |  | 0.23*** | (0.02) | 0.23*** | (0.02) |
| School climate - Fair norms |  |  |  |  |  |  | 0.02 | (0.02) | 0.02 | (0.02) |
| School climate - Student participation |  |  |  |  |  |  | 0.17*** | (0.02) | 0.17*** | (0.02) |
| Peer victimization - Threats |  |  |  |  |  |  | 0.00 | (0.02) | 0.01 | (0.02) |
| Peer victimization - physical |  |  |  |  |  |  | -0.01 | (0.01) | -0.01 | (0.01) |
| Peer victimization - sexual |  |  |  |  |  |  | 0.04 | (0.02) | 0.03 | (0.02) |
| Peer victimization - verbal |  |  |  |  |  |  | -0.00 | (0.01) | -0.00 | (0.01) |
| Perceived teachers' well-being |  |  |  |  |  |  | 0.21*** | (0.02) | 0.21*** | (0.02) |
| Teacher-to-student victimization |  |  |  |  |  |  | 0.01 | (0.01) | 0.00 | (0.01) |
| *School-level* |  |  |  |  |  |  |  |  |  |  |
| *School vulnerability index (reference: Low-vulnerability school)* | | | | | | | | | | |
| Medium-vulnerability school |  |  |  |  |  |  |  |  | -0.01 | *(0.03)* |
| High-vulnerability school |  |  |  |  |  |  |  |  | 0.04 | *(0.02)* |
| School climate - Total (school average) |  |  |  |  |  |  |  |  | 0.13 | (0.08) |
| Peer victimization - Total (school average) |  |  |  |  |  |  |  |  | 0.04** | (0.01) |
| Perceived teachers' well-being (school average) |  |  |  |  |  |  |  |  | -0.03 | (0.06) |
| Teacher-to-student victimization (school average) |  |  |  |  |  |  |  |  | -0.01 | (0.06) |
| Variance components | Null Model | | Model 1 | | Model 2 | | Model 4 | | Model 6 | |
| Student-level variance | 0.633 | (0.014) | 0.628 | (0.014) | 0.546 | (0.012) | 0.327 | (0.010) | 0.327 | (0.010) |
| School-level variance | 0.052 | (0.008) | 0.025 | (0.006) | 0.02 | (0.005) | 0.006 | (0.002) | 0.004 | (0.001) |
| % Level 1 variance explained | Base | | 0.68% | | 13.69% | | 48.4% | | 48.37% | |
| % Level 2 variance explained | Base | | 50.99% | | 60.42% | | 87.71% | | 92.96% | |

Note: Standardized coefficients reported. Standard errors in parentheses. Explained variance compared to null model. **p* < .05, ***p* < .01, ****p* <.001

# Supplementary Table 2.

*Summary of multi-level linear regression analysis for variables predicting social acceptance at school at individual and school level (N=6,389 students at 212 schools)*

|  | Null Model | | Model 1 | | Model 2 | | Model 3 | | Model 4 | |
| --- | --- | --- | --- | --- | --- | --- | --- | --- | --- | --- |
| Variables | *b* | (SE) | *b* | (SE) | *b* | (SE) | *b* | (SE) | *b* | (SE) |
| Constant | -0.00 | (0.02) | 0.72*** | (0.11) | 0.38** | (0.12) | -0.68*** | (0.15) | -0.90** | (0.33) |
| *Student-level* |  |  |  |  |  |  |  |  |  |  |
| Gender (Female=1) |  |  | 0.06** | (0.02) | 0.07** | (0.02) | 0.05* | (0.02) | 0.05* | (0.02) |
| Age |  |  | -0.04*** | (0.01) | -0.04*** | (0.01) | -0.03*** | (0.01) | -0.02*** | (0.01) |
| Number of ICTs |  |  | -0.02*** | (0.00) | -0.02*** | (0.00) | -0.02*** | (0.00) | -0.02*** | (0.00) |
| Family satisfaction |  |  |  |  | 0.04*** | (0.00) | -0.01** | (0.00) | -0.01** | (0.00) |
| Change of schools |  |  |  |  | -0.04** | (0.01) | -0.01 | (0.01) | -0.01 | (0.01) |
| 2018 GPA |  |  |  |  |  |  | 0.06*** | (0.01) | 0.06*** | (0.01) |
| Friends satisfaction |  |  |  |  |  |  | 0.08*** | (0.02) | 0.07*** | (0.02) |
| School climate - Teachers social support |  |  |  |  |  |  | 0.13*** | (0.02) | 0.13*** | (0.02) |
| School climate - Fair norms |  |  |  |  |  |  | 0.08*** | (0.02) | 0.08*** | (0.02) |
| School climate - Student participation |  |  |  |  |  |  | 0.04 | (0.02) | 0.04 | (0.02) |
| Peer victimization - Threats |  |  |  |  |  |  | -0.04* | (0.02) | -0.04* | (0.02) |
| Peer victimization - physical |  |  |  |  |  |  | 0.02 | (0.01) | 0.02 | (0.01) |
| Peer victimization - sexual |  |  |  |  |  |  | -0.03 | (0.02) | -0.03 | (0.02) |
| Peer victimization - verbal |  |  |  |  |  |  | -0.04*** | (0.01) | -0.04*** | (0.01) |
| Perceived teachers' well-being |  |  |  |  |  |  | 0.07*** | (0.02) | 0.06** | (0.02) |
| Teacher-to-student victimization |  |  |  |  |  |  | -0.05*** | (0.01) | -0.05*** | (0.01) |
| *School-level* |  |  |  |  |  |  |  |  |  |  |
| *School vulnerability index (reference: Low-vulnerability school)* | | | | | | | | | | |
| Medium-vulnerability school |  |  |  |  |  |  |  |  | -0.00 | *(0.03)* |
| High-vulnerability school |  |  |  |  |  |  |  |  | -0.01 | *(0.03)* |
| School climate - Total (school average) |  |  |  |  |  |  |  |  | 0.10 | (0.10) |
| Peer victimization - Total (school average) |  |  |  |  |  |  |  |  | -0.04* | (0.02) |
| Perceived teachers' well-being (school average) |  |  |  |  |  |  |  |  | 0.02 | (0.06) |
| Teacher-to-student victimization (school average) |  |  |  |  |  |  |  |  | -0.10 | (0.08) |
| Variance components |  | |  | |  | |  | |  | |
| Student-level variance | 0.604 | (0.017) | 0.597 | (0.017) | 0.589 | (0.017) | 0.521 | (0.018) | 0.522 | (0.017) |
| School-level variance | 0.049 | (0.008) | 0.042 | (0.007) | 0.039 | (0.007) | 0.019 | (0.004) | 0.012 | (0.004) |
| % Level 1 variance explained | Base | | 1.16% | | 2.48% | | 13.74% | | 13.58% | |
| % Level 2 variance explained | Base | | 14.29% | | 20.41% | | 61.22% | | 75.51% | |

Note: Standardized coefficients reported. Standard errors in parentheses. Explained variance compared to null model. **p* < .05, ***p* < .01, ****p* <.001

# Supplementary Table 3.

*Summary of multi-level linear regression analysis for variables predicting social actualization at school at individual and school level (N=6,389 students at 212 schools)*

|  | Null Model | | Model 1 | | Model 2 | | Model 3 | | Model 4 | |
| --- | --- | --- | --- | --- | --- | --- | --- | --- | --- | --- |
| Variables | *b* | (SE) | *b* | (SE) | *b* | (SE) | *b* | (SE) | *b* | (SE) |
| Constant | 0.02 | (0.02) | 0.80*** | (0.12) | -0.17 | (0.12) | -2.56*** | (0.12) | -3.21*** | (0.24) |
| *Student-level* |  |  |  |  |  |  |  |  |  |  |
| Gender (Female=1) |  |  | 0.06** | (0.02) | 0.09*** | (0.02) | 0.06** | (0.02) | 0.06** | (0.02) |
| Age |  |  | -0.06*** | (0.01) | -0.05*** | (0.01) | -0.02*** | (0.01) | -0.01* | (0.01) |
| Number of ICTs |  |  | 0.00 | (0.00) | 0.00 | (0.00) | -0.00 | (0.00) | -0.00 | (0.00) |
| Family satisfaction |  |  |  |  | 0.11*** | (0.01) | 0.02*** | (0.00) | 0.02*** | (0.00) |
| Change of schools |  |  |  |  | 0.01 | (0.01) | 0.01 | (0.01) | 0.01 | (0.01) |
| 2018 GPA |  |  |  |  |  |  | 0.01 | (0.01) | 0.01 | (0.01) |
| Friends satisfaction |  |  |  |  |  |  | 0.12*** | (0.02) | 0.12*** | (0.02) |
| School climate - Teachers social support |  |  |  |  |  |  | 0.08*** | (0.02) | 0.07*** | (0.02) |
| School climate - Fair norms |  |  |  |  |  |  | 0.09*** | (0.02) | 0.09*** | (0.02) |
| School climate - Student participation |  |  |  |  |  |  | 0.13*** | (0.02) | 0.13*** | (0.02) |
| Peer victimization - Threats |  |  |  |  |  |  | 0.00 | (0.02) | 0.00 | (0.02) |
| Peer victimization - physical |  |  |  |  |  |  | -0.01 | (0.01) | -0.01 | (0.01) |
| Peer victimization - sexual |  |  |  |  |  |  | 0.02 | (0.02) | 0.01 | (0.02) |
| Peer victimization - verbal |  |  |  |  |  |  | 0.00 | (0.01) | 0.00 | (0.01) |
| Perceived teachers' well-being |  |  |  |  |  |  | 0.44*** | (0.01) | 0.44*** | (0.02) |
| Teacher-to-student victimization |  |  |  |  |  |  | 0.01 | (0.01) | 0.01 | (0.01) |
| *School-level* |  |  |  |  |  |  |  |  |  |  |
| *School vulnerability index (reference: Low-vulnerability school)* | | | | | | | | | | |
| Medium-vulnerability school |  |  |  |  |  |  |  |  | 0.04 | (0.03) |
| High-vulnerability school |  |  |  |  |  |  |  |  | 0.01 | (0.02) |
| School climate - Total (school average) |  |  |  |  |  |  |  |  | 0.28*** | (0.08) |
| Peer victimization - Total (school average) |  |  |  |  |  |  |  |  | 0.01 | (0.01) |
| Perceived teachers' well-being (school average) |  |  |  |  |  |  |  |  | -0.08 | (0.05) |
| Teacher-to-student victimization (school average) |  |  |  |  |  |  |  |  | 0.04 | (0.06) |
| Variance components |  | |  | |  | |  | |  | |
| Student-level variance | 0.658 | (0.015) | 0.654 | (0.014) | 0.607 | (0.013) | 0.385 | (0.011) | 0.385 | (0.011) |
| School-level variance | 0.059 | (0.009) | 0.034 | (0.006) | 0.029 | (0.005) | 0.003 | (0.001) | 0.001 | (0.001) |
| % Level 1 variance explained | Base | | 0.61% | | 7.75% | | 41.49% | | 41.49% | |
| % Level 2 variance explained | Base | | 42.37% | | 50.85% | | 94.92% | | 98.31% | |

Note: Standardized coefficients reported. Standard errors in parentheses. Explained variance compared to null model. **p* < .05, ***p* < .01, ****p* <.001

# Supplementary Table 4.

*Summary of multi-level linear regression analysis for variables predicting social coherence at school at individual and school level (N=6,389 students at 212 schools)*

|  | Null Model | | Model 1 | | Model 2 | | Model 3 | | Model 4 | |
| --- | --- | --- | --- | --- | --- | --- | --- | --- | --- | --- |
| Variables | *b* | (SE) | *b* | (SE) | *b* | (SE) | *b* | (SE) | *b* | (SE) |
| Constant | 0.00 | (0.02) | -0.11 | (0.10) | 0.10 | (0.11) | 0.93*** | (0.16) | 1.24*** | (0.28) |
| *Student-level* |  |  |  |  |  |  |  |  |  |  |
| Gender (Female=1) |  |  | -0.10*** | (0.02) | -0.11*** | (0.02) | -0.07*** | (0.02) | -0.07*** | (0.02) |
| Age |  |  | 0.00 | (0.01) | -0.00 | (0.01) | -0.01 | (0.01) | -0.01 | (0.01) |
| Number of ICTs |  |  | 0.01*** | (0.00) | 0.01*** | (0.00) | 0.01*** | (0.00) | 0.01*** | (0.00) |
| Family satisfaction |  |  |  |  | -0.03*** | (0.00) | 0.00 | (0.00) | 0.00 | (0.00) |
| Change of schools |  |  |  |  | 0.06*** | (0.01) | 0.03** | (0.01) | 0.03* | (0.01) |
| 2018 GPA |  |  |  |  |  |  | -0.10*** | (0.01) | -0.09*** | (0.01) |
| Friends satisfaction |  |  |  |  |  |  | -0.04* | (0.02) | -0.03 | (0.02) |
| School climate - Teachers social support |  |  |  |  |  |  | -0.08*** | (0.02) | -0.08*** | (0.02) |
| School climate - Fair norms |  |  |  |  |  |  | -0.05** | (0.02) | -0.05** | (0.02) |
| School climate - Student participation |  |  |  |  |  |  | -0.00 | (0.02) | -0.00 | (0.02) |
| Peer victimization - Threats |  |  |  |  |  |  | 0.08*** | (0.02) | 0.08*** | (0.02) |
| Peer victimization - physical |  |  |  |  |  |  | -0.01 | (0.01) | -0.01 | (0.01) |
| Peer victimization - sexual |  |  |  |  |  |  | 0.03 | (0.02) | 0.03 | (0.02) |
| Peer victimization - verbal |  |  |  |  |  |  | 0.01 | (0.01) | 0.01 | (0.01) |
| Perceived teachers' well-being |  |  |  |  |  |  | -0.01 | (0.02) | -0.01 | (0.02) |
| Teacher-to-student victimization |  |  |  |  |  |  | 0.07*** | (0.01) | 0.07*** | (0.01) |
| *School-level* |  |  |  |  |  |  |  |  |  |  |
| *School vulnerability index (reference: Low-vulnerability school)* | | | | | | | | | | |
| Medium-vulnerability school |  |  |  |  |  |  |  |  | 0.04 | (0.03) |
| High-vulnerability school |  |  |  |  |  |  |  |  | 0.06 | (0.03) |
| School climate - Total (school average) |  |  |  |  |  |  |  |  | -0.13 | (0.09) |
| Peer victimization - Total (school average) |  |  |  |  |  |  |  |  | -0.03* | (0.02) |
| Perceived teachers' well-being (school average) |  |  |  |  |  |  |  |  | -0.02 | (0.05) |
| Teacher-to-student victimization (school average) |  |  |  |  |  |  |  |  | -0.05 | (0.07) |
| Variance components |  | |  | |  | |  | |  | |
| Student-level variance | 0.511 | (0.015) | 0.505 | (0.015) | 0.500 | (0.015) | 0.464 | (0.015) | 0.464 | (0.015) |
| School-level variance | 0.028 | (0.005) | 0.027 | (0.005) | 0.024 | (0.004) | 0.014 | (0.003) | 0.009 | (0.003) |
| % Level 1 variance explained | Base | | 1.17% | | 2.15% | | 9.20% | | 9.20% | |
| % Level 2 variance explained | Base | | 3.57% | | 14.29% | | 50.00% | | 67.86% | |

Note: Standardized coefficients reported. Standard errors in parentheses. Explained variance compared to null model. **p* < .05, ***p* < .01, ****p* <.001
